# Supplementary figures and images for: The Physiotherapy Process of a Plegic Patient Who Communicates with Foot Movement—A Case Report
Source: Brain Sci. 2022 May 25;12(6):688. doi: 10.3390/brainsci12060688 (PMC9220889; doi:10.3390/brainsci12060688)

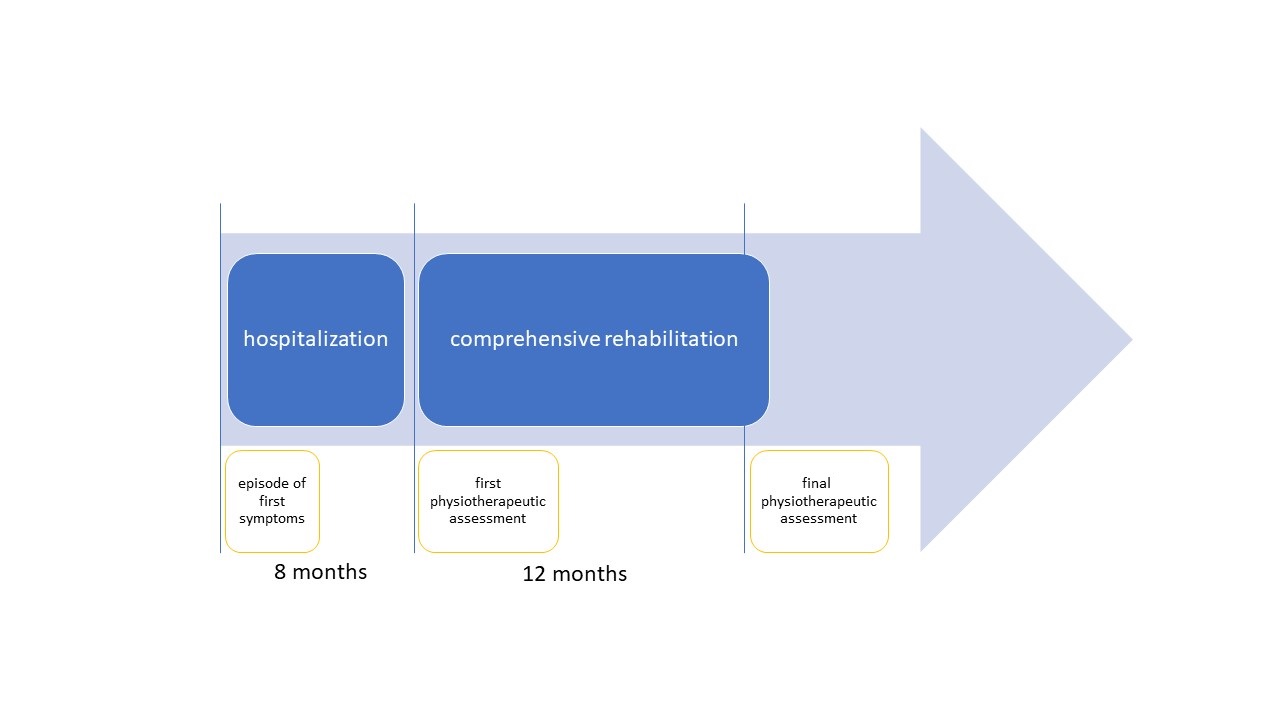

Supplement: Supplementary file 1 [file brainsci-12-00688-s001.zip › brainsci-1706839-supplementary.jpg]
